# Drivers and magnitude of food insecurity among rural households in southern Democratic Republic of Congo

**DOI:** 10.1016/j.heliyon.2024.e40207

**Published:** 2024-11-07

**Authors:** Victor Manyong, Paul Martin Dontsop Nguezet, Dieu-Merci Akonkwa Nyamuhirwa, Romanus Osabohien, Mpoko Bokanga, Jacob Mignouna, Zoumana Bamba, Razack Adeoti

**Affiliations:** a Department of Scocial Science and Agribusiness, International Institute of Tropical Agriculture (IITA), Dar es salaam, Tanzania; b Department of Scocial Science and Agribusiness, International Institute of Tropical Agriculture (IITA), MbujiMayi, Democratic Republic of the Congo; c Institute of Agricultural Policy and Market Research, Justus Liebig University Giessen (JLU), Senckenbergstraße 3, 35390, Gießen, Giessen, Germany; d Universiti Tenega Nastional (UNITEN), Kajang, Malaysia; e Fondation- Plantations et Huileries du Congo (PHC), Kinshasa, Democratic Republic of the Congo; f Campus Olusegun Obasanjo, International Institute of Tropical Agriculture (IITA), Bukavu, Democratic Republic of the Congo; g Central Africa Hub, International Institute of Tropical Agriculture (IITA), Kinshasa, Democratic Republic of the Congo; h Department of Scocial Science and Agribusiness, International Institute of Tropical Agriculture (IITA), Cotonou, Benin

**Keywords:** Food insecurity, Food diversity and scarcity, Negative binomial model, Rural households, Kasai Oriental province, Democratic Republic of Congo

## Abstract

Access to adequate and nutritious food is accepted as a human right worldwide. In the Democratic Republic of Congo (DRC), Kasai Oriental province is one of the most vulnerable provinces in the country in terms of food insecurity. However, its current depth of food insecurity and the root factors have not been studied. Against this background, this study used cross-sectional data from 318 households to analyze the magnitude and socioeconomic drivers of food insecurity among rural households in the province. We developed two food security indicators: the food consumption score (FCS) and household food insecurity access scale (HFIAS), and applied the ordinary least square (OLS) and the negative binomial model for the analysis. Our findings show that households rely mainly on vegetables to meet their food need, consume more energy, and have limited access to protein, vitamin, and fat-rich foods. All the surveyed households were deficient in food quantity, while 75 % were deficient in quality. The study noted that the severity of food insecurity is zone-specific and more pronounced in the Kabeya Kamwanga territory than in others. Importantly, the poverty and education levels among households associated with large household sizes were the significant determinants of food insecurity in the area. These results strongly demonstrate the need for agrifood interventions that foster education, enable efficient land use, and target poor households in the province.

## 1 Introduction

Adequate access to nutritious food for all humans will remain at the top of government and the international community's priorities for an extended period as it has been a human right since 2000 [[1], [2], [3]]. This fact is encapsulated in the United Nations (UN) Sustainable Development Goals (SDGs), particularly SDG1 of Eliminating Poverty and SDG2 of Zero Hunger and Malnutrition. Conceptually, food security implies “physical and economic access to sufficient safe and nutritious foods to meet one's dietary needs and food preferences for an active and healthy life” [[4], [5], [6], [7], [8]]. Therefore, healthy nutrition significantly contributes to human body development [9,10].

As the global population continues to grow in the face of climate change, providing sufficient food, that is affordable, nutritious, and safe remains a challenge for all countries in the world [11]. While countries of the world strive for the speedy actualization of food security and zero poverty goals, these efforts are hindered by other socioeconomic shocks such as the recent COVID-19 pandemic, the Russian–Ukrainian war, and global inflation, which have upset progress toward these goals worldwide [[12], [13], [14]], making the availability of and access to food for billions of people more uncertain [[15], [16], [17], [18], [19], [20]]. The FAO et al. [18] food insecurity report shows that almost 3.2 billion people worldwide cannot afford a healthy diet, among which almost 828 million are hungry. The most recent food security update by the World Bank [21] mentions the Democratic Republic of Congo (DRC), among the few countries in the world, as a hotspot of very high concern for significant levels of acute food insecurity.

Malnutrition prevents children from reaching their full physical and mental potential [22,23]. It contributes to the high prevalence of various diseases, including diabetes and cardiovascular risk factors such as hypertension for adolescents and adults [24]. Being the world's second most food-insecure country, the DRC accounts for 26 million people in acute food insecurity, and 5 million children are malnourished [25]. The poverty rate is about 73 %, and food production has decreased by almost 60 % since independence [[25], [26], [27], [28]]. As mentioned above, the country's economic perspectives have been slowed down by the recent crises, especially the COVID-19 pandemic [28]. Furthermore, some provinces, such as Kasai Oriental, need emergency interventions, while others do not and perform relatively better [29,30].

DRC has excellent agricultural and climatic conditions. In addition, the government has initiated many national agricultural programs to revitalize the agricultural sector and improve the population's food security since the 1990s [31,32]. Tshiebue [27] counted more than 15 public agriculture programs initiated up to 2017 (see Table A1). Despite these efforts, there is still a gap between the present state of agriculture production and food security and policy responses from these interventions [33]. This gap is attributed more significantly to political instability, local violence, health crises, and agricultural constraints that prevented productivity increase and access to the market, but rarely to agriculture policy design and implementation [21,34].

Some provinces have received less attention from researchers leading to a lack of information in designing adequate agriculture policies [30,35]. In 2019, the Global Data Lab estimated the poverty rate at 99 % among the population in Kasai Oriental province, which was the highest in the country compared to other provinces [35]. However, less is known in terms of the depth and drivers of food insecurity in this area. This study, therefore, contributes to the existing literature by assessing the magnitude and drivers of food insecurity among rural households in the Kasai Oriental province of DRC. Specifically, it answers two research questions. First, what is the status of food insecurity among rural households in Kasai Oriental province? Second, what are the socioeconomic drivers of food insecurity?

After this introductory section, the remainder of the paper proceeds as follows: Section 2 presents the methodology; Section 3 presents the results and discussion, Section 4 presents the results of the robustness check of econometric analyses, and Section 5 concludes, presents policy implications and limitations.

## 2 Methodology

### 2.1 Study area and data

Data for this study were collected in October 2020 in Kasai Oriental province using a multistage data collection process. Located in southern DRC, Kasai Oriental province is known for diamond mining located in Mbuji-Mayi, its capital city. However, this province is also among the most densely populated provinces in DRC, with about 6 million people depending on agriculture and livestock as other sources of income [36]. Dense forests characterize the northern part of Kasai Oriental, while savanna-like landscapes characterize the south of the province [36].

**Fig. 1:**
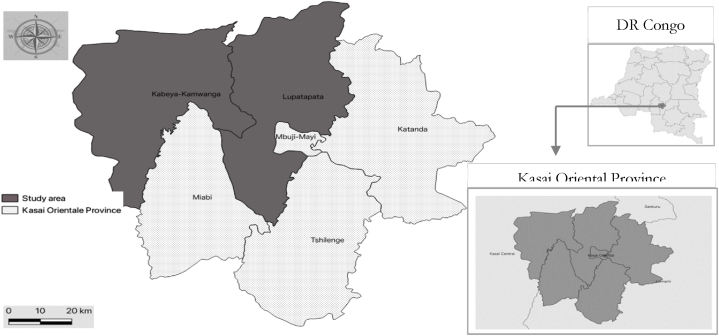
Maps showing the study area. Notes. Authors' construct using QGIS.

Out of the five (5) territories that make up Kasai Oriental province, Kabeya Kamwanga and Lupatapata have, on average less access to education (e.g., the number of schools per village) and health services (e.g., the rate of prenatal consultations), coupled with a poor state of infrastructure compared to other territories. A multistage sampling approach was used in data collection. In the first stage, we purposively selected these two territories with poor access to education and health services (Fig. 1). It is worth noting that Lupatapata had better access to water and low average prices for essential basic food items such as maize and cassava compared to Kabeya Kamwanga [36].

In the second stage, we purposively selected one sector out of four in each of the two territories based on their population density: Ndomba in Kabeya Kamwanga, where many activities could be developed because of the presence of the port and Mukumbi in Lupatapata. In the third stage, villages from the two selected sectors were listed and ranked based on their importance (low or high) in maize or cassava production and their level of accessibility, out of which a total of 33 villages were chosen.

The sample size was determined by using the following formula [37]:

(1) $${N=p\left(1-p\right)\left(z|\epsilon \right)}^{2}$$

where N is the sample size; z is the statistic for a level of confidence of 95 %, which is (1.96); ϵ is the sampling error (level of precision), which was 5 %, and p is the approximative proportion of the population living in the rural areas, which is 68 % for this study. By using the formula in Eq. (1), the final sample size was 334 households, representing about 10 households per village. At the village level, households were selected using a systematic sampling approach starting from the main road in each village. After cleaning data, 318 households were retained for data analyses.

For data collection, the field team relied on an electronically designed and programmed survey to capture critical features such as respondent characteristics (age, sex, education, etc.), household characteristics (household size, cultivated land, or farming experience), and food security indicators, geographic location, etc. After pretesting, the electronic questionnaire was administered using semistructured interviews by trained enumerators with the minimum level of secondary school education and who fluently speak and understand both French and Tshiluba (the most popular local language used in Kasai Oriental province).

Enumerators administered the questionnaire in French and Tshiluba when and where needed. ODK enabled timely data aggregation on the Ona server (https://ona.io), where quality control was immediately performed, and possible errors were reported to the supervisor for onsite correction before the enumerators moved from one village to the next. The electronic questionnaire's food and nutritional security module contained data on seven days' food consumption recall, capturing the dietary diversity of the household, and one month's consumption behavior recall, capturing access to food and food scarcity in the household.

### 2.2 Empirical approach

Guided by the nature of food security indicators used in this study–food consumption score (FCS), household food insecurity access scale (HFIAS), and household food insecurity access prevalence (HFIAP) [38], on the one hand, and their relevance as recommended by the existing literature, on the other hand [7,38,39], this paper used the ordinary least square (OLS), and the negative binomial model to identify socioeconomic and demographic drivers of food insecurity.

The FCS is an indicator reflecting the quantity and quality of food consumed by a household during a given period [40]. Therefore, it reflects food availability and utilization dimensions of food security. It is captured using a seven-day recall of eight food groups and corresponds to the sum of the products of the consumption frequencies and the weightings associated with each food group. Empirically, the FCS is obtained as expressed in Eq. (2):

(2) $$FCS=\sum_{g=1}^{8}\left(f_{g}*\varphi_{g}\right)$$

Where $f_{g}$ is the observed frequency of consumption of each food group g over seven days preceding the survey; $\varphi_{g}$ is the weight associated with each food group g given its nutritional contribution to the diet and FCS the food consumption score [39,40]. Three categories can be derived from the continuous FCS following Marivoet et al. [39] and Kennedy et al. [40]. Following these studies, the rule of thumb stipulates that an FCS below 21.5 is associated with poor consumption status, between 21.5 and 35 is associated with limited food consumption, and above 35, the household is considered to have acceptable food consumption.

The quantitative and continuous nature of the FCS is compatible with the OLS model. Therefore, the OLS model was selected among other multiple regression models because the study considered the food consumption score as a linear function of a vector of household h socioeconomic and demographic variables $Z_{h}$, and respondent i characteristics, $X_{i}$ taken as control variables (Eq. (3)). The empirical model was specified as follows.

(3) $${FCS}_{h}=\zeta_{ih}+\omega Z_{h}+\theta {X_{i}+\mu }_{ih}$$

The HFIAS captures access to food. Gebreyesus et al. [34] assumed that “the experience of food insecurity (access) causes predictable reactions and responses that can be quantified and summarized in a scale.” Therefore, HFIAS is an account outcome variable. This indicator has been applied in several social science studies to account for household food access [41]. Based on specific questions, the HFIAS score is obtained from categorical variables reflecting food scarcity within households for 30 days, ranging between 0 and 27. The observed values of HFIAS do not have any quantitative meaning. For example, zero is the HFIAS value corresponding to all the households who answer “no” to all the nine questions and are assumed to be food secure.

Considering the nature of this account outcome variable and the need for great flexibility in model fitting, we used a negative binomial model following Tuholske et al. [7] and Chang [42].

Furthermore, considering the nature of the FCS categories and the HFIAP, we performed a multinomial logit model to estimate the probability of observing each categorical outcome of the FCS (1 = acceptable, 2 = limited 3 = poor) and HFIAP (1 = Food secure access, 2 = Mild food-insecure access, 3 = Moderate food-insecure access, and 4 = Severe food-insecure access). The fact that the average HFIAS score is a continuous variable means that it is much more sensitive to detecting more minor changes over time than the HFIAP indicator. Therefore, the HFIAP indicator was reported in addition to the HFIAS average score because it overcomes this sensitivity and provides a good measure for monitoring the intervention [38].

The multinomial logit (mlogit) model is an extension of the binary logit model used when the dependent variable has multiple categories that are not ordered [43]. The observed outcome of the FCS and HFIAP categories ($y_{ih}$) in household h is given in Eq. (4):

(4) $$y_{ih}=\zeta_{ih}+\omega Z_{ih}+\theta {X_{ih}+\mu }_{ih}$$

Where Z and X are household and household head characteristics that affect food security, respectively. We estimated Eq. (4) using the mlogit Stata command.

### 2.3 Household and respondent characteristics

*PPI Score:* The assessment of household poverty conditions relied on the poverty probability index (PPI). The PPI score was based on ten questions about a household's characteristics and asset ownership. Based on an international poverty line of US$1.9/person/day, the PPI score derives a likelihood of the respondent's household being below the poverty line [44]. The final indicator is a numeric variable of the probability of being poor. This study relied on the validated procedure for Rwanda for two main reasons [44]. First, DRC and Rwanda are in the same category (low category) regarding human development index and human capital index. Second, the poverty rate trend in both countries has been similar since 2010, so too have their PPI procedures [28,45]. Following previous studies, we expect the PPI score to be negatively associated with food insecurity [7,20,21,46,47].

*Cultivated land*: As Capaldo et al. [48] have noted, it can be argued that land in rural areas remains an essential resource for two reasons. First, because it guarantees income from rents, and second, because land ownership guarantees access to credit; this would imply that the larger the cultivated area, the less likely a household is to be food insecure. However, when it comes to cultivating lands, it has been argued, following the studies of Olasehinde-Williams et al. [49] and Holden and Gherbu [50], that in the absence of mechanization, smaller, cultivated areas of land yield good outcomes as, on average, they require less labor, time, and effort to be managed compared to larger areas, hence we may hypothesis that smaller areas of land may be positively associated with food security.

*Higher education*: As emphasized by Manda et al. [51] and Nyamuhirwa et al. [52], education as human capital comes into play in treating information about agricultural technologies, understanding their importance, and hence their utilization. Therefore, households with a member educated to the highest level may know a difference compared to uneducated households such that their food status may be better than the latter [7,53]. Education was captured in terms of the level of education following the DRC education system.

*Household size*: The relationship between household size and food security is relevant, first, because larger households are associated with higher food needs in terms of quantity, and second, the tradeoff between quantity and quality makes larger households more likely to be food insecure [20]. Therefore, we hypothesize that a larger household size is associated with a low food consumption score and may experience more anxiety related to food insecurity [20,[54], [55], [56]]. The household size included all people who live together and eat out of the same pot as follows: someone who has temporarily moved for less than six months, students studying away from home, workers who have stayed for at least a month, and someone who lives away from home but is very involved in household economic decision-making. Finally, other household and respondent characteristics were taken as exogenous factors, including the age of the respondent, the sex of the respondent, and the respondent's farming experience. The territory the respondent resides in was used as a control variable.

## 3 Results and discussion

### 3.1 Socioeconomic characteristics of respondents

Table 1 shows that a large majority (76 %) of household heads were male, and more than 60 % of them were less than 50 years of age. Most of the respondents (90 %) attained a primary education level. However, the average number of people living in the same household was relatively high (10 persons). The respondents have agriculture as their primary source of livelihood. Also, they have a relatively small land area, generally less than 2 ha, and around 60 % have more than ten years of farming experience. Using the PPI, the households had a 50 % chance to live below the national poverty line of US$1.9/person/day. Households in Lupatapata were less likely to be poor compared to Kabeya Kamwanga, 39 % and 51 %, respectively, implying that, on average, Lupatapata households had a higher standard of living compared to Kabeya Kamwanga households.

**Table 1:** Descriptive statistics of variables of interest.

Variables	Territories				Overall sample	
	Kabeya Kamwanga		Lupatapata			
	Mean	Std. Err.	Mean	Std. Err.	Mean	Std. Err.
PPI Score∗∗∗	35.6	0.5	39.7	0.7	37.8	0.4
	N	Percent	N	Percent	N	Percent

Cultivated land∗∗∗						
Less than 2 ha	111	74.0 %	84	50.6 %	195	61.7 %
Between 2 and 5 ha	30	20.0 %	75	45.2 %	105	33.2 %
More than 5 ha	9	6.0 %	7	4.2 %	16	5.0 %
Level of education						
Analphabet	15	10.0 %	17	9.5 %	31	9.8 %
Primary	85	56.7 %	97	57.7 %	182	57.2 %
Secondary	48	32.0 %	51	30.4 %	99	31.1 %
University	2	1.3 %	3	2.0 %	5	1.9 %
Household size						
1–6	31	20.7 %	55	32.7 %	86	27.0 %
7–12	76	50.7 %	76	45.2 %	152	47.8 %
13–18	34	22.7 %	29	17.3 %	63	19.8 %
More than 18	9	6.0 %	8	4.8 %	17	5.4 %
Sex						
Male	113	75.3 %	128	76.2 %	241	75.8 %
Female	37	24.7 %	40	23.8 %	77	24.2 %
Age∗∗∗						
18–30	31	20.7 %	34	20.2 %	65	20.4 %
31–40	46	30.7 %	31	18.5 %	77	24.2 %
41–50	19	12.7 %	49	29.2 %	68	21.4 %
51–60	33	22.0 %	36	21.4 %	69	21.7 %
More than 60	21	14.0 %	18	10.7 %	39	12.3 %
Farming experience∗∗∗						
1–10	48	33.1 %	75	47.5 %	123	40.6 %
11–20	30	20.7 %	36	22.8 %	66	21.8 %
21–30	39	26.9 %	25	15.8 %	64	21.1 %
More than 30	28	19.3 %	22	13.9 %	50	16.5 %
Observations	150		168		318	

Notes. Significant at ∗∗∗*p* < 0.01, ∗∗*p* < 0.05, ∗*p* < 0.1. PPI = Progress out of poverty index.

### 3.2 Households’ food diversity and scarcity

Table 2 shows the food groups consumed by households in the seven days before the survey and the frequency of consumption. The results show that three food groups were the most consumed, including vegetables (98 %), cereal and tubers (95 %), and sweet products such as sugar, honey, candies, and others (sweetened beverages) (72 %). Vegetables were consumed on average six days per week, while cereal, tubers, and sweets were consumed 5 and 4 days per week. These results suggest that households consume more dietary energy and very little protein, vitamins, and fat-rich foods, hence low-quality food in general. These results can be explained by the underdeveloped livestock, fishery, and aquaculture sectors, on the one hand, and the dependency of the province on imported food, which is the primary cause of the fluctuations in food availability and prices. For example, the fish consumed in Kasai Oriental province are imported from Kinshasa, Lubumbashi, and Kalemie and from abroad, including Namibia, Angola, Zambia, Malawi, and Tanzania. However, transit in other provinces increases transaction costs, leading to higher consumer prices. The rearing of large and small livestock such as cows, pigs, and sheep is negligible.

**Table 2:** Household food diversity.

	Food group consumed			Frequency of consumption of food group		
	Kabeya Kamwanga	Lupatapata	Overall	Kabeya Kamwanga	Lupatapata	Overall
Vegetables	95.33	99.40	97.48	6.26	6.52	6.40
Cereal and tuber	94.67	95.83	95.28	4.01	5.70	4.90
Sweet products	62.67	80.95	72.33	2.91	4.98	4.00
Dried vegetables	38.67	63.10	51.57	1.33	2.52	1.96
Spices, condiments, and beverages	41.33	48.81	45.28	1.49	2.61	2.08
Fruit	37.33	33.93	35.53	1.31	1.37	1.34
Oils and fats	11.33	28.57	20.44	0.81	2.20	1.55
Meat and fish	4.67	20.24	12.89	0.05	0.42	0.25
Milk and milk products	0.67	2.38	1.57	0.01	0.03	0.02

**Fig. 2:**
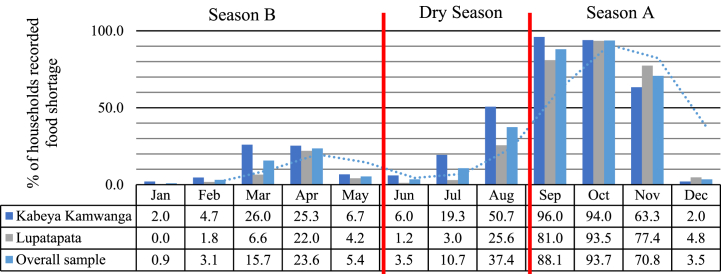
Households' food scarcity periods.

Moreover, the assessment of households’ food scarcity shows two stressful periods associated with the two cropping seasons: A – September to January and B – February to May (Fig. 2). The critical months when more than 70 % of households record food scarcity are September, October, and November. This period of the year is characterized by food shortages and increased food prices [36]. This situation is mainly linked to poor postharvest handling of agriculture products, including storage, which increases postharvest losses and the difficulty in accessing Kasai Oriental from other provinces due to the poor state of road infrastructure coupled with severe agricultural constraints that prevent productivity increases and access to the markets [36,57]. These analyses are significant for policy design in two aspects. First, they shed light on the stressful months for the local population, and second, they demonstrate the need for investment in agricultural production, storage facilities and improvements of road infrastructure to sustainably address the issue of food scarcity in Kasai Oriental province.

### 3.3 Food security assessment

**Fig. 3:**
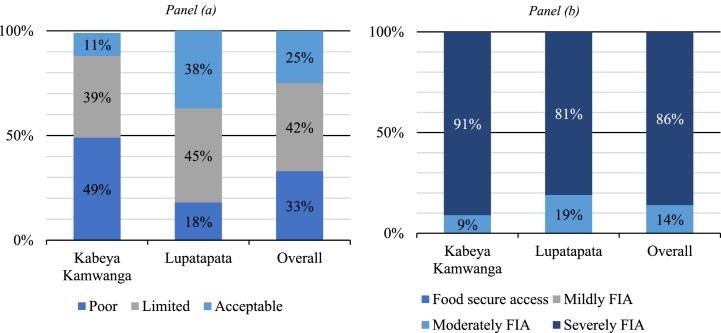
Distribution of households by FCS categories in panel (a) and by HFIAP categories in panel (b). Note. FIA denotes food insecurity access.

This section focuses on assessing food security based on two indicators, FCS and HFIAS, and their respective categories (Fig. 3). Considering FCS, over 75 % of households were categorized as food insecure given their poor or limited food consumption (Fig. 3, panel (a)). This situation was more pronounced in Kabeya Kamwanga, where about 89 % of households were food insecure in both quantity and quality of food consumed, while in Lupatapata, the prevalence was relatively low and stood at around 63 %.

While the FCS is estimated using data from the seven-day recall approach, the HFIAP, however, uses data covering four weeks; it gives more insight into the ability of a typical household to access preferred and sufficient safe and nutritious foods. The HFIAP indicator results showed that all the surveyed households had poor access to food. About 86 % of households were classified as moderately food insecure, while 14 % were severely food insecure. Using this indicator, the severity of food insecurity was still consistently more pronounced in Kabeya Kamwanga than in Lupatapata (Fig. 3, panel (b)).

The difference in the proportion between the two territories by each food indicator category was significant using the chi-square (χ^2^) statistic. These results align with the study of Marivoet et al. [29,39] in DRC. They can be explained by the poor picture of the province characterized by recent political instability, poverty, illiteracy, poor housing quality, little access to electricity and water, and roads in disrepair as painted by the Global Data Lab [33] and UNHCR, [58].

**Fig. 4:**
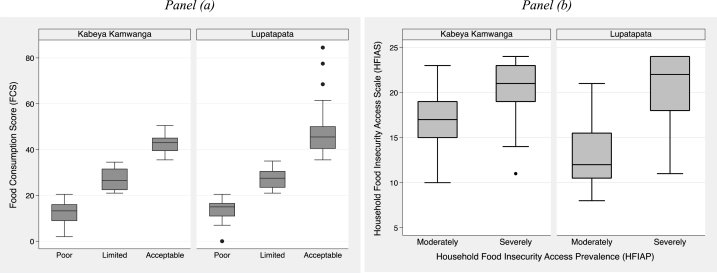
Box plot of FCS and FCS categories in Panel (a) and HFIAS and HFIAP categories in Panel (b).

Fig. 4, panels (a) and (b), assess the relationship between each food indicator (FCS and HFIAS) and its corresponding categories. Higher FCS corresponds to acceptable food consumption, and a lower HFIAS corresponds to higher access to preferred, sufficient, and nutritious food. The HFIAS mean values were 16.5 and 20.9, respectively, for moderately and severely food-insecure access in Kabeya Kamwanga territory. The combined mean value was 20.3. In Lupatapata, the HFIAS mean values were 12.9 and 20.3 for moderately and severely food-insecure access, with a combined mean of 18.9. The mean differences were statistically significant in both territories at 1 % level (t-stat = 5 and t-stat = 10, respectively).

### 3.4 Assessment of drivers of food security

Table 3, Table 4 present the results of socioeconomic drivers of food security considering both FCS, HFIAS, and FCS and HFIAP categories. Table 3 presents the ordinary least square and negative binomial results, while Table 4 presents the multinomial logit. The OLS models are satisfactory, following the F statistics indicating a good fitness of the models and the LR statistics for the negative binomial regression and multinomial logit. Besides, the use of multiple specifications provides consistent results across models.

**Table 3:** Determinants of food consumption score and household insecurity access scale.

Variables	Food Consumption Score			Household Food Insecurity Access Scale		
	Ordinary Least Square (OLS)			Negative Binomial Model		
	(1)	(2)	(3)	(4)	(5)	(6)
	Coef.	Coef.	Coef.	IRR	IRR	IRR
PPI score	0.64(0.086) ∗∗∗	0.50(0.108) ∗∗∗	0.48(0.109) ∗∗∗	−0.01(0.002) ∗∗∗	−0.01(0.002) ∗∗∗	−0.01(0.002) ∗∗∗
Cultivated land						
Less than 2 ha (ref.)		1	1		1	1
Between 2 and 5 ha		0.82(1.549)	0.41(1.553)		−0.10(0.031) ∗∗∗	−0.09(0.031) ∗∗∗
More than 5 ha		−7.58(3.004) ∗∗	−7.94(2.999) ∗∗∗		−0.06(0.060)	−0.07(0.060)
Highest level of education						
Analphabet (ref.)		1	1		1	1
Primary		7.95(2.304) ∗∗∗	8.69(2.323) ∗∗∗		−0.07(0.045)	−0.08(0.046) ∗
Secondary		7.24(2.314) ∗∗∗	8.01(2.345) ∗∗∗		−0.02(0.045)	−0.04(0.046)
University		11.11(3.951) ∗∗∗	12.37(3.986) ∗∗∗		0.03(0.080)	0.02(0.081)
Household size						
1–6 (ref.)		1	1		1	1
7–12		0.34(1.571)	0.59(1.600)		0.00(0.031)	−0.01(0.032)
13–18		0.44(1.973)	1.05(2.055)		−0.01(0.039)	−0.04(0.041)
More than 18		−6.44(3.139) ∗∗	−6.19(3.265) ∗		0.11(0.062) ∗	0.07(0.065)
Control	No	No	Yes	No	No	Yes
Territory	Yes	Yes	Yes	Yes	Yes	Yes
Observations	318	318	318	318	318	318
F stat. and LR	58.95∗∗∗	16.14∗∗∗	13.03∗∗∗	42.6∗∗∗	64.7∗∗∗	71.0∗∗∗

Notes. Ref. = category of reference. Coef. = coefficient. Significant at ∗∗∗*p* < 0.01, ∗∗*p* < 0.05, ∗*p* < 0.1. Model (1) is a simple OLS assessing the relationship between food security and poverty. In model (2), the effect of poverty on food security is controlled by household characteristics. In models (3) and (6), respondent characteristics (age, sex, and farming experience) are considered. Parentheses contain normal standard errors. The constant is not reported.

**Table 4:** Probability models of FCS categories and HFIAP.

Variables	FCS categories						HFIAP		
	Multinomial Logit						Multinomial Logit		
	(1)	(2)	(4)	(5)	(6)	(7)	(8)	(9)	(10)
	Poor	Limited	Poor	Limited	Poor	Limited	Moderately	Moderately	Moderately
	Coef.	Coef.	Coef.	Coef.	Coef.	Coef.	Coef.	Coef.	Coef.
PPI score	−0.147∗∗∗	−0.054∗∗∗	−0.102∗∗∗	−0.031	−0.097∗∗∗	−0.029	0.102∗∗∗	0.111∗∗∗	0.109∗∗∗
	(0.027)	(0.018)	(0.035)	(0.025)	(0.035)	(0.025)	(0.020)	(0.026)	(0.027)
Cultivated land 2–5 ha			−0.404	−1.167∗∗∗	−0.318	−1.204∗∗∗		1.192∗∗∗	1.199∗∗∗
			(0.422)	(0.362)	(0.430)	(0.368)		(0.410)	(0.417)
Education level									
Primary			−2.377∗∗∗	−1.983∗∗	−2.533∗∗∗	−1.967∗∗		0.846	0.822
			(0.901)	(0.877)	(0.913)	(0.886)		(0.873)	(0.884)
Secondary			−2.158∗∗	−1.717∗∗	−2.385∗∗∗	−1.795∗∗		0.071	0.124
			(0.906)	(0.876)	(0.923)	(0.886)		(0.870)	(0.884)
University			−4.074∗∗∗	−2.636∗∗	−4.703∗∗∗	−2.803∗∗		−0.144	−0.156
			(1.500)	(1.146)	(1.537)	(1.188)		(1.211)	(1.239)
Household size									
7–12			0.383	0.520	0.240	0.437		0.821	0.874∗
			(0.438)	(0.370)	(0.453)	(0.383)		(0.521)	(0.530)
13–18			0.376	0.264	0.188	0.158		1.319∗∗	1.410∗∗
			(0.551)	(0.480)	(0.585)	(0.505)		(0.588)	(0.614)
More than 18			2.481∗∗	2.286∗∗	2.353∗∗	2.100∗∗		−0.358	−0.203
			(1.091)	(0.970)	(1.139)	(1.002)		(0.924)	(0.958)
Control	No	No	No	No	Yes	Yes	No	No	Yes
Territory	Yes	Yes	Yes	Yes	Yes	Yes	Yes	Yes	Yes
Observations	318	318	318	318	318	318	318	318	318
LR	86.1∗∗∗	133.2∗∗∗	142.9∗∗∗	34.8∗∗∗	60.0∗∗∗	60.4∗∗∗			

Notes. Coef. = coefficient. The reference FCS category is the “acceptable” category, while for HFIAP, the reference category is “severely Food Insecurity Access.” Significant at ∗∗∗*p* < 0.01, ∗∗*p* < 0.05, ∗*p* < 0.1. Models (1), (2), and (8) linked food security and poverty by controlling territory. In models (6), (7), and (10), respondent characteristics (age, sex, and experience) are taken into account in addition to household characteristics. Parentheses contain standard errors. The constant is not reported.

The assessment of households’ poverty conditions relied on the PPI score following Desiere et al. [44] and Manyong et al. [20]. Table 3 shows that the PPI score was significantly associated with food adequacy measured by the FCS and food access based on HFIAS, suggesting that the less a household was likely to be poor, the higher its food availability and access. Concretely, an increase of 10 points in PPI score led to an increase in FCS of 6.4 points, while the percent change in the incident rate of HFIAS was a 0.01 % decrease for every unit increase in PPI score (Table 4—Models (1) and (4), respectively, for FCS and HFIAS).

Besides, the likelihood of being poor or having limited food adequacy decreased by 0.147 and 0.054, and the likelihood of being moderately food insecure access increased by 0.102 for each point increase in PPI score (Table 4—Models (1), (2), and (8)). This result was comparable to the results of the studies by Rossi et al. [22], Maitra and Rao [46], Tuholske et al. [7], and Manyong et al. [20], which found that poverty was strongly associated with food and nutrition security, with low agricultural production as a common denominator, especially in rural areas. Moreover, Kasai Oriental province has the highest poverty rate (99 %) in the country compared to other provinces [35].

Cultivated land is vital in agriculture as it is the main base for food production. The analysis below demonstrates that cultivated land of less than 5 ha significantly increased access to food, and when it exceeds 5 ha, it decreased household food adequacy (Table 3). An increase in land cultivated reduces household likelihood of being food insecure in terms of food adequacy and food access (Table 4). Possible explanations could be related to mechanization, on the one hand, and land property rights, on the other. Smaller cultivated lands are better managed than large land cultivated without mechanization, as they have lower labor, time, and effort demands, as Olasehinde-Williams et al. [49] found in 25 sub-Saharan African countries. However, weak land property rights could limit farmers’ investments, such as mechanization and irrigation, often associated with high food production and access, as evidenced by Holden and Gherbu [50]. Land property rights remain problematic as it is a dominant source of land conflict in the study area, as shown by UNHCR [57]. Similarly, as Lipton and Saghai [58] and Baral et al. [53] argue, better land reforms and higher access to land are followed by improvements in food and nutritional security.

The role of education in rural areas is undeniable. The highest educational attainment of the household head was found to increase household food adequacy significantly, but education was not a significant predictor of food access (Table 3, Table 4). Moreover, households with at least one member who attended or completed primary, secondary, and tertiary education had a higher associated FCS (Table 3) and a lower likelihood of food insecurity (Table 4). The magnitude of these effects was pronounced for households with at least one member with a university education level. As shown by Tuholske et al. [7] and Baral et al. [53], education was associated with fewer anxieties related to the inability to access food and high nutrition intake.

Household size and food security are inversely related. This relationship implies a tradeoff between the quantity and quality of consumed food, making larger households more vulnerable to food insecurity [20]. Table 3 shows that the larger the household size (more than 18 household members), the lower its FCS, indicating less food availability. Besides, larger household size was associated with a higher likelihood of food insecurity (Table 4). These findings were in line with those of Bhalla et al. [54], who found that larger household size was associated with a lower value of per capita food consumption in Zimbabwe, and those of Nguezet et al. [55], who found that larger families were open to buy affordable new food products due to budget constraints. However, there was no consistent significant effect of household size on HFIAS while it significantly increased the likelihood of moderately food insecure access, as in the study by Broussard [56].

## 4 Robustness check

Since a reverse causality could exist between poverty and food security, we first computed the Pearson correlation to test the magnitude of the association between PPI score and food security indicators. The associated Pearson correlation coefficient was significantly lower, indicating a lower correlation between the PPI score and FCS (0.44) on the one hand and the PPI score and HFIAS (−0.38) on the other. Moreover, the PPI score does not incorporate income, food consumption, and expenditures, which are normally and theoretically linked directly to food security and may accentuate the endogeneity issue [16,44,59].

Furthermore, various alternative model specifications were performed for robustness check following Lu and White's [58] definition of robustness check. The relation between PPI score and food security remained robust and consistent in all the models when controlling for household and household head characteristics (Table 3, Table 4). The robustness check analysis presented in Table 5, Table 6 considered only household and respondent characteristics and omitted the PPI score. The findings showed that the variables that significantly explained food security “with PPI score” in Table 3, Table 4 were the same “without PPI score” in Table 4, Table 5 However, a two-stage least squares (2SLS) could resolve the endogeneity issue consistently. Still, the identification of the instrument for poverty concerning the exclusion restriction assumption was limited by our dataset. For example, Maitra and Rao [46] suggested using household labor type as an instrument to address this limitation. The justification of this instrument is that households that rely only on casual labor are more vulnerable to poverty; hence, poverty may affect food security only through the type of labor.

**Table 5:** Determinants of food consumption score and household insecurity access scale.

Variables	Food Consumption Score		Household Food Insecurity Access Scale	
	(1)	(2)	(3)	(4)
	Coef.	Coef.	IRR	IRR
Cultivated land (ref. = less than 2 ha)				
Between 2 and 5 ha	2.403(1.561)		−0.127(0.030) ∗∗∗	
More than 5 ha	−5.301(3.063) ∗		−0.104(0.059) ∗	
Education level				
Primary	12.534(2.152) ∗∗∗		−0.160(0.040) ∗∗∗	
Secondary	13.094(2.010) ∗∗∗		−0.132(0.037) ∗∗∗	
University	20.555(3.505) ∗∗∗		−0.158(0.068) ∗∗	
Household size (ref. = less than 7)				
7–12	−0.353(1.616)		0.015(0.031)	
13–18	0.218(2.037)		−0.009(0.039)	
More than 18	−4.040(3.199)		0.063(0.061)	
Sex (ref. = male)		−2.315(1.689)		0.023(0.030)
Household head age		0.005(0.060)		−0.000(0.001)
Household head farming experience		−0.053(0.060)		0.003(0.001) ∗∗
Territory	Yes	Yes	Yes	Yes
Observations	318	318	318	318

Notes. Ref. = category of reference. Coef. = coefficient. Significant at ∗∗∗*p* < 0.01, ∗∗*p* < 0.05, ∗*p* < 0.1. Models (1) and (3) include household characteristics, while Models (2) and (4) consider respondent characteristics. Parentheses contain normal standard errors. The constant is not reported. Models (3) and (4) are negative binomial, while Models (1) and (2) are OLS.

**Table 6:** Probability models of FCS categories and HFIAP.

Variables	FCS categories				HFIAP	
	(1)	(2)	(3)	(4)	(5)	(6)
	Poor	Limited	Poor	Limited	Moderately	Moderately
	Coef.	Coef.	Coef.	Coef.	Coef.	Coef.
Cultivated land (ref. = less than 2 ha)						
Between 2 and 5 ha	−0.659(0.409)	−1.266(0.350) ∗∗∗			1.567(0.385) ∗∗∗	
More than 5 ha	0.426(0.849)	−1.018(0.824)			−0.241(1.119)	
Education level						
Primary	−3.368(0.842) ∗∗∗	−2.319(0.831) ∗∗∗			2.025(0.809) ∗∗	
Secondary	−3.353(0.817) ∗∗∗	−2.087(0.807) ∗∗∗			1.605(0.779) ∗∗	
University	−6.278(1.390) ∗∗∗	−3.074(1.007) ∗∗∗			1.951(1.026) ∗	
Household size (ref. = less than 7)						
7–12	0.473(0.429)	0.488(0.363)			0.425(0.468)	
13–18	0.327(0.534)	0.214(0.463)			0.885(0.533) ∗	
More than 18	1.808(0.998) ∗	1.744(0.909) ∗			0.341(0.798)	
Sex (ref. = male)			0.401(0.372)	−0.147(0.349)		0.140(0.374)
Household head age			0.007(0.013)	0.001(0.012)		0.006(0.013)
Household head farming experience			0.007(0.014)	0.013(0.013)		−0.016(0.014)
Territory	Yes	Yes	Yes	Yes	Yes	Yes
Observations	318	318	318	318	318	318

Notes. Ref. = category of reference. Coef. = coefficient. Significant at ∗∗∗*p* < 0.01, ∗∗*p* < 0.05, ∗*p* < 0.1. Models (1) and (3) include household characteristics, while Models (2) and (4) consider respondent characteristics. Parentheses contain normal standard errors. The constant is not reported. All the models are multinomial logit.

## 5 Conclusion, policy implications, and limitations

This study analyzed the magnitude and the socioeconomic determinants of food insecurity among rural households in Kasai Oriental province using two indicators of food security, one for each dimension (availability and accessibility). Overall, the food status of surveyed households needs to be improved in quantity and quality. Regarding food adequacy, 75 % of households were food insecure, and all the surveyed households were found to be food insecure regarding access, demonstrating that access to sufficient, nutritious, and preferred food is an emergency problem in this province. In addition, the severity of food insecurity was more pronounced in the Kabeya Kamwanga territory than in Lupatapata.

We found three main drivers regarding food insecurity: high poverty, low education, and large household size, while cultivated land increased food security. Food consumption improvements were driven by a significant improvement in the PPI score, which showed how fighting food insecurity remains primarily linked to poverty considerations. This result indicates that the lesser the likelihood of a rural household being poor, the better its food status. Education remains a strong channel of food security improvement, especially regarding food adequacy. However, we did not find any significant effect on food access. This result reinforces that food security is not only about knowledge but also about the economic capability of rural households to access decent food.

Indeed, not all drivers of food security can improve food adequacy and food access at the same time. In addition to education, this study shows that the household size at some level (more than 18 household members) significantly improves food adequacy but reduces access insignificantly. At the same time, a smaller number of household members (less than 18 members) improves access to food. This result stresses the tradeoff between quantity and food quality regarding household size. Moreover, this study has demonstrated that the smaller the cultivated land, the more access to food, meaning that supporting farmers in terms of mechanization, for example, could lead to better management of large land cultivated. Finally, it is not evident that food abundance is followed by good food utilization, so there is a relevant need for food education.

Based on the findings from this study, three policy recommendations can be formulated. First, access to land and more land security should be one of the priorities at the local level. Second, implementing large interventions that are market-oriented, improving agricultural feeder roads, and improving access to suitable agricultural inputs and knowledge improves the value of locally produced products and addresses the limits of households in food utilization. Third, there is a need to design a sustainable framework to monitor and implement food and nutrition-sensitive interventions.

Although the findings of this study provide relevant policy recommendations for addressing food insecurity in Kasai Oriental province, it has two limitations. First, it relies on cross-sectional data and does not provide a longitudinal analysis of food security to reflect on the stability dimension of food security. Second, although our analyses do not present a problem of endogeneity, a Two-Stage Least Square (2SLS) should still be considered in future studies to ensure the endogeneity issue is systematically and consistently addressed.

## Consent to participateauthor-disclosure

All participants in the study gave their informed consent.

## CRediT authorship contribution statement

**Victor Manyong:** Writing – review & editing, Supervision, Funding acquisition, Conceptualization. **Paul Martin Dontsop Nguezet:** Writing – review & editing, Investigation, Conceptualization, Supervision. **Dieu-Merci Akonkwa Nyamuhirwa:** Writing – review & editing, Writing – original draft, Visualization, Supervision, Software, Methodology, Investigation, Formal analysis, Data curation, Conceptualization. **Romanus Osabohien:** Writing – review & editing. **Mpoko Bokanga:** Writing – review & editing, Funding acquisition, Project administration. **Jacob Mignouna:** Writing – review & editing. **Zoumana Bamba:** Writing – review & editing. **Razack Adeoti:** Writing – review & editing, Supervision, Investigation, Data curation, Conceptualization.

## Data availability statement

Data will be made available on request.

## Ethics approval

Permission to conduct this study was approved by the International Institute of Tropical Agriculture Internal Review Board (IRB) number IRB/001/2024.

## Funding statement

The 10.13039/100022898International Institute of Tropical Agriculture (IITA) funded this study under project number PJ3384 from a grant by Fonds pour la Promotion de l’Industrie (FPI).

## Declaration of competing interest

The authors declare that they have no known competing financial interests or personal relationships that could have appeared to influence the work reported in this paper.

## Figures and Tables

**Table A1:** DR Congo's Agriculture Sector Public Initiatives since 1966.

N°	Programs	Period
1	Plan intérimaire de relance agricole	1966–1972
2	Plan Mobutu	1979–1981
3	Programme Agricole Minimum	1980
4	Plan de Relance Agricole	1982–1984
5	Programme Intérimaire de Réhabilitation Économique	1983–1985
6	Plan Quinquennal	1986–1990
7	Programme d’Autosuffisance alimentaire	1987–1990
8	Plan directeur du Développement agricole et rural	1991–2000
9	Programme national de Relance du Secteur agricole et rural	1997–2001
10	Programme triennal minimum	1997–1999
11	Programme triennal d’Appui aux Producteurs du Secteur agricole	2000–2003
12	Programme multisectoriel d’Urgence, de Réhabilitation et de Reconstruction	2002–2010
13	Programme minimum de Partenariat pour la Transition et la Relance « PMPTR » en RDC	2004–2005
14	Document de la Stratégie de Croissance et de Réduction de la Pauvreté (DSCRP); version intérimaire en 2004, DSCRP1 en 2006, DSCRP nouvelle génération en 2011	Since 2004
15	Note de Politique agricole	2009
16	Stratégie sectorielle de l’Agriculture et du Développement rural	2010
17	Loi portant principes fondamentaux relatifs à l’Agriculture	2011
18	Plan national d’Investissement agricole PNIA	2013–2020
19	Programme National Stratégique de Développement (PNSD)	2019–2023
20	Plan Provincial d’Investissement Agricole	2020–2023
21	Programme de Développement Local des 145 Territoire	2021–2023
22	Agenda de Transformation Agricole	2022–2023

## Abbreviations

FCS: Food Consumption Score
FPI: Fonds pour la Promotion de l’Industrie
HFIAP: Household Food Insecurity Access Prevalence
HFIAS: Household Food Insecurity Access Scale
IITA: International Institute of Tropical Agriculture
PPI: Poverty Probability Index
UNHCR: The UN Refugee Agency
