# Supplementary material for: Drivers and magnitude of food insecurity among rural households in southern Democratic Republic of Congo
Source: Heliyon. 2024 Nov 7;10(21):e40207. doi: 10.1016/j.heliyon.2024.e40207 (PMC11838079; doi:10.1016/j.heliyon.2024.e40207)
Supplement: Multimedia component 1 [file mmc1.pdf]

Kasai_Study

Section 1. Identification

Je travaille pour l'IITA et le FPI, et suis chargé de conduire la présente enquête avec la collaboaration de l'INERA au nom de la RDC. L'objectif de l'étude est de collecter les informations sur la situation actuelle des producteurs pour le développement de Chaînes de Valeurs Agricoles prioritaires, pouvant contribuer au développement du secteur agricole et rural de la région par la promotion et l'émergence d'opérateurs économiques dans le secteur agroalimentaire. Vos réponses à nos questions seront gardées de façon confidentielle et utilisées seulement pour le but de la recherché sans faire référence à votre nom.

Avant de commencer par administrer les questions, l'objectif de l'étude doit être lu par l'enquêteur et bien traduit /expliqué au producteur

---

N ° d'identification du questionnaire

L'identifiant doit inclure les deux initiales du nom de l'enquêteur suivi du numero du ménage, Ex. L'enquêteur Akonkwa Dieu-Merci mettra : Ak001, Ak002, etc. pour ménage 1 et 2, etc.

Nom de l'intervieweur

- ○ Pierrot MUKA
- ○ Leon TSHISUAKA
- ○ Jean Pierre TSHILUMBA
- ○ Charles BADINENGANYI

Insérez la province

- ○ Kasai Oriental

Insérez le Territoire

- ○ Kabeya Kamuanga
- ○ Lupatapata

Insérez le secteur

- ○ Ndomba
- ○ Mukumbi

Insérez le groupement

- ○ Mpanda
- ○ Bakua Kanjinga
- ○ Bena Mpeta
- ○ BAJILA KASANGA II
- ○ BAKWA MULUMBA
- ○ BAKWA LUKOKA

Insérez le village (Ndomba)

- BNA KABAMBA II
- BENA MULOMBO
- BENA KABAMBA I
- BENA NGELEKA
- BENA KAZADI TSHIBIAYI
- BENA KAZADI LUMBAYI
- BAKUA KASHILA
- BENA MBUYI II
- BENA MBUYI I
- BENA BITULU
- BENA MBUYAMBA
- BENA KAPONGO
- BENA TSHIPANDA
- BENA MUPOMPA
- BENA NKUMBI
- BAKUA NSANGAMAYI
- BAKUA NAPITA
- BENA LUKANU
- BAKUA KANYINDA
- BENA MUTOMBO

Insérez le village (Mukumbi)

- BK NSAKALONGA
- BK BISAMPU
- BK ISBE
- BK MBUYI
- BK KABALA
- BK TORONTO
- BK MULUMBA
- BK INCENDIE
- BK BABILEMBI
- BK DIJIBA
- BK POSITION
- BK BISAMPUKA
- BK KANKELENGE
- BK TUTSULU
- BK MABAYA
- BK BIBOTA
- BK KATULAYI
- BK TSHILUNDE
- BK LUKOKA
- BK NYANGA

N ° du ménage

Le répondant est-il le chef de ménage?

- Oui
- Non

Si non, affiliation au chef de ménage

L'Interview doit être réalisée avec l'un des deux adultes du ménage i.e. mari ou épouse

- Epouse
- Mari
- Enfant adulte habitant dans la maison

Numéro de téléphone des répondants ou des amis ou pareents plus proches

Mettez 99 si le répondant n'a pas de numéro de téléphone

Coordonnées GPS

latitude (x.y °)

longitude (x.y °)

altitude (m)

accuracy (m)

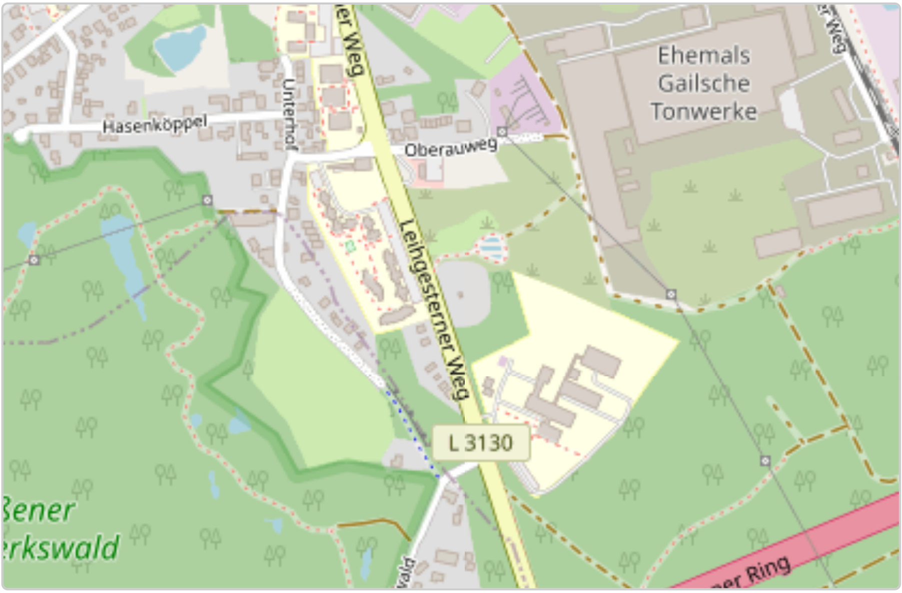

Date de l'entretien

Seule la date de l'entretien est acceptable. Tachez donc de finaliser votre formulaire le jour même de l'entretien sinon vous serez obligé de changer la date pour le finaliser.

yyyy-mm-dd

Heure de début d'enquete

hh:mm

Section 2. Informations générales

Nom de l'enquêté(e)

Sexe de l'enquêté(e)

- ○ Homme
- ○ Femme

Âge de l'enquêté(e)

Statut matrimonial de l'enquêté(e)

- ○ célibataire
- ○ Marié(e)
- ○ Divorcé(e)
- ○ Veuf(ve)
- ○ Autre

Autre, à preciser.

Niveau d'éducation académique du chef de ménage

- ○ Aucune
- ○ Primaire
- ○ Secondaire
- ○ Universitaire
- ○ Informelle/alphabétisation
- ○ Autre

Autre, à preciser.

Niveau Education le plus élevé atteint dans le ménage

- ○ Aucune
- ○ Primaire
- ○ Secondaire
- ○ Universitaire
- ○ Informelle/alphabétisation
- ○ Autre

Autre, à preciser.

Nombre d'hommes âgés de plus de 10 ans

Nombre de femmes âgées de plus de 10 ans

Nombre de membres en dessous de 10 ans

Nombre de membres total dans le ménage

Expérience du chef de ménage en agriculture? (nombre d'années)

Principales sources de revenus du chef de ménage

- ☐ Agriculture
- ☐ Transformation
- ☐ Commerce
- ☐ Élevage
- ☐ Pecheur
- ☐ Autre

Autre, à preciser.

Le chef de ménage est-il membre d'une association?

- ○ Oui
- ○ N'est plus
- ○ Jamais

Type d'associations

- organisation paysanne
- Platform
- association culturelle
- association politique
- association religieuse
- ONG
- Autre

Autre, à preciser.

Quelle a été votre motivation pour adhérer à l'association?

- accès facile au crédit
- L'approvisionnement en maïs par l'association
- Transport collectif de marchandises/maïs
- Vente Groupée de maïs
- Accès facile aux intrants
- Autre

Autre, à preciser.

Combien de temps avez-vous été membre (en années)?

Votre statut de membre au sein de l'association

- Membre Simple
- Responsible Bureau
- Autre

Autre, à preciser.

Est-ce l'association fonctionne encore?

- Oui
- Non

Quels avantages tirez-vous de votre appartenance à l'association /groupement?

- Accès facile au crédit
- L'approvisionnement en maïs par l'association
- Transport collectif de marchandises/maïs
- Vente Groupée de maïs
- Accès facile aux intrants
- Autre

Autre, à preciser.

Section 3. Sécurité alimentaire et nutritionnelle

» Section 3.1 Sources de revenu du ménage

Quelles est la principale sources de revenu du ménage ?

- Ventes de production végétale
- Ventes de la production animale
- Produits de ressources naturelles
- Emploi permanent formel
- Travail occasionnel
- Travaux contractuels semi-qualifiés
- Entreprise / activité génératrice de revenus
- Ventes d'actifs
- Location de terrains
- Cadeaux / envois de fonds
- Pension
- Employé comme locataire
- Pêche
- Carré minier
- Autre

Autre, à preciser.

Hormis la principale source de revenus, quelle était la source secondaire de revenus de votre ménage entre Octobre 2019 et septembre 2020?

- Ventes de production végétale
- Ventes de la production animale
- Produits de ressources naturelles
- Emploi permanent formel
- Travail occasionnel
- Travaux contractuels semi-qualifiés
- Entreprise / activité génératrice de revenus
- Ventes d'actifs
- Location de terrains
- Cadeaux / envois de fonds
- Pension
- Employé comme locataire
- Pêche
- Carré minier
- Autre

Autre, à preciser.

» Section 3.2 Disponibilite et Consommation d'aliments

Combien de fois mangez-vous normalement par jour?

- une
- deux
- trois fois
- plus de trois

Y a-t-il des mois dans l'année pendant lesquels votre maison connaît normalement des pénuries alimentaires ?

- Oui
- Non

Si oui, priere indiquer les mois

- Janvier
- Fevrier
- Mars
- Avril
- Mai
- Juin
- Juillet
- Aout
- Septembre
- Octobre
- Novembre
- Decembre

» Section. 3.3 Diversité alimentaire des ménages / score de consommation alimentaire

Prière fournir les informations sur les achats des produits alimentaires effectués dans les 7 derniers jours

Votre ménage a-t-il consommé cette nourriture au cours des sept derniers jours?

Céréales: Tout nshima, bouillie, pain, riz, biscuits, nouilles ou tout aliment à base de maïs, riz, blé, millet, sorgho ou tout autre grain

- Oui
- Non

Nombre de JOURS de consommation au cours des 7 derniers jours?

Principale source de nourriture consommée

- dons sociaux (faits par la communauté / la famille ou des individus)
- propre production
- crédit / prêt
- parents / amis / voisins
- pêche / chasse / cueillette
- Dons humanitaires (faits par l'état et / ou des organisations)
- paiement du travail
- achat
- Autre

Votre ménage a-t-il consommé cette nourriture au cours des sept derniers jours?

Racines et tubercules: patates douces blanches ou jaunes, pommes de terre irlandaises, igname, manioc blanc ou d'autres aliments issus de racines?

- Oui
- Non

Nombre de JOURS de consommation au cours des 7 derniers jours?

Principale source de nourriture consommée

- dons sociaux (faits par la communauté / la famille ou des individus)
- propre production
- crédit / prêt
- parents / amis / voisins
- pêche / chasse / cueillette
- Dons humanitaires (faits par l'état et / ou des organisations)
- paiement du travail
- achat
- Autre

Votre ménage a-t-il consommé cette nourriture au cours des sept derniers jours?

Légumineuses, noix et graines - haricots secs, des arachides ou d'autres aliments fabriqués à partir de ceux-ci (par exemple, le beurre d'arachide)?

- Oui
- Non

Nombre de JOURS de consommation au cours des 7 derniers jours?

Principale source de nourriture consommée

- dons sociaux (faits par la communauté / la famille ou des individus)
- propre production
- crédit / prêt
- parents / amis / voisins
- pêche / chasse / cueillette
- Dons humanitaires (faits par l'état et / ou des organisations)
- paiement du travail
- achat
- Autre

Votre ménage a-t-il consommé cette nourriture au cours des sept derniers jours?

Légumes à feuilles vert foncé : Légumes à feuilles vert foncé, y compris des formes sauvages et des feuilles riches en vitamine A disponibles localement telles que l'amaranthe, les feuilles de manioc, les feuilles de citrouille, les feuilles de patate douce, le chou frisé, les épinards, le gombo?

- Oui
- Non

Nombre de JOURS de consommation au cours des 7 derniers jours?

Principale source de nourriture consommée

- dons sociaux (faits par la communauté / la famille ou des individus)
- propre production
- crédit / prêt
- parents / amis / voisins
- pêche / chasse / cueillette
- Dons humanitaires (faits par l'état et / ou des organisations)
- paiement du travail
- achat
- Autre

Votre ménage a-t-il consommé cette nourriture au cours des sept derniers jours?

Autres legumes : Toutes les tomates, les oignons

- Oui
- Non

Nombre de JOURS de consommation au cours des 7 derniers jours?

Principale source de nourriture consommée

- dons sociaux (faits par la communauté / la famille ou des individus)
- propre production
- crédit / prêt
- parents / amis / voisins
- pêche / chasse / cueillette
- Dons humanitaires (faits par l'état et / ou des organisations)
- paiement du travail
- achat
- Autre

Votre ménage a-t-il consommé cette nourriture au cours des sept derniers jours?

Fruit riche en vitamine A: Toute mangue mûre, papaye mûre

- Oui
- Non

Nombre de JOURS de consommation au cours des 7 derniers jours?

Principale source de nourriture consommée

- dons sociaux (faits par la communauté / la famille ou des individus)
- propre production
- crédit / prêt
- parents / amis / voisins
- pêche / chasse / cueillette
- Dons humanitaires (faits par l'état et / ou des organisations)
- paiement du travail
- achat
- Autre

Votre ménage a-t-il consommé cette nourriture au cours des sept derniers jours?

Autres fruits : gpyaves, oranges, avocat, y compris des fruits sauvages

- Oui
- Non

Nombre de JOURS de consommation au cours des 7 derniers jours?

Principale source de nourriture consommée

- ◯ dons sociaux (faits par la communauté / la famille ou des individus)
- ◯ propre production
- ◯ crédit / prêt
- ◯ parents / amis / voisins
- ◯ pêche / chasse / cueillette
- ◯ Dons humanitaires (faits par l'état et / ou des organisations)
- ◯ paiement du travail
- ◯ achat
- ◯ Autre

Votre ménage a-t-il consommé cette nourriture au cours des sept derniers jours?
Viande de chair - bœuf, porc, agneau, chèvre, gibier, crocodile, poulet, canard, pintade, pigeon, caille ou autres oiseaux, insectes?

- ◯ Oui
- ◯ Non

Nombre de JOURS de consommation au cours des 7 derniers jours?

____

Principale source de nourriture consommée

- ◯ dons sociaux (faits par la communauté / la famille ou des individus)
- ◯ propre production
- ◯ crédit / prêt
- ◯ parents / amis / voisins
- ◯ pêche / chasse / cueillette
- ◯ Dons humanitaires (faits par l'état et / ou des organisations)
- ◯ paiement du travail
- ◯ achat
- ◯ Autre

Votre ménage a-t-il consommé cette nourriture au cours des sept derniers jours?
Abats - foie, reins, cœur ou autres abats ou des aliments à base de sang?

- ◯ Oui
- ◯ Non

Nombre de JOURS de consommation au cours des 7 derniers jours?

____

Principale source de nourriture consommée

- ◯ dons sociaux (faits par la communauté / la famille ou des individus)
- ◯ propre production
- ◯ crédit / prêt
- ◯ parents / amis / voisins
- ◯ pêche / chasse / cueillette
- ◯ Dons humanitaires (faits par l'état et / ou des organisations)
- ◯ paiement du travail
- ◯ achat
- ◯ Autre

Votre ménage a-t-il consommé cette nourriture au cours des sept derniers jours?
Poisson - poisson frais ou séché (par exemple kapenta, dorade, chisense, etc.) ?

- Oui
- Non

Nombre de JOURS de consommation au cours des 7 derniers jours?

Principale source de nourriture consommée

- dons sociaux (faits par la communauté / la famille ou des individus)
- propre production
- crédit / prêt
- parents / amis / voisins
- pêche / chasse / cueillette
- Dons humanitaires (faits par l'état et / ou des organisations)
- paiement du travail
- achat
- Autre

Votre ménage a-t-il consommé cette nourriture au cours des sept derniers jours?
Œufs : œufs de poule, canard, pintade, crocodile

- Oui
- Non

Nombre de JOURS de consommation au cours des 7 derniers jours?

Principale source de nourriture consommée

- dons sociaux (faits par la communauté / la famille ou des individus)
- propre production
- crédit / prêt
- parents / amis / voisins
- pêche / chasse / cueillette
- Dons humanitaires (faits par l'état et / ou des organisations)
- paiement du travail
- achat
- Autre

Votre ménage a-t-il consommé cette nourriture au cours des sept derniers jours?
Lait et autres produits laitiers: Lait frais / caillé, yogourt, fromage, autres produits laitiers (Lait en poudre: seulement si des verres de lait

- Oui
- Non

Nombre de JOURS de consommation au cours des 7 derniers jours?

Principale source de nourriture consommée

- dons sociaux (faits par la communauté / la famille ou des individus)
- propre production
- crédit / prêt
- parents / amis / voisins
- pêche / chasse / cueillette
- Dons humanitaires (faits par l'état et / ou des organisations)
- paiement du travail
- achat
- Autre

Votre ménage a-t-il consommé cette nourriture au cours des sept derniers jours?
Huiles et graisses - Toutes les huiles, graisses ou beurre ajoutés aux aliments ou préparés pour la cuisson

- Oui
- Non

Nombre de JOURS de consommation au cours des 7 derniers jours?

Principale source de nourriture consommée

- dons sociaux (faits par la communauté / la famille ou des individus)
- propre production
- crédit / prêt
- parents / amis / voisins
- pêche / chasse / cueillette
- Dons humanitaires (faits par l'état et / ou des organisations)
- paiement du travail
- achat
- Autre

Votre ménage a-t-il consommé cette nourriture au cours des sept derniers jours?
Bonbons - sucre, miel?

- Oui
- Non

Nombre de JOURS de consommation au cours des 7 derniers jours?

Principale source de nourriture consommée

- dons sociaux (faits par la communauté / la famille ou des individus)
- propre production
- crédit / prêt
- parents / amis / voisins
- pêche / chasse / cueillette
- Dons humanitaires (faits par l'état et / ou des organisations)
- paiement du travail
- achat
- Autre

Votre ménage a-t-il consommé cette nourriture au cours des sept derniers jours?
Epices, condiments, boissons - Des épices, du café, du thé, des boissons alcoolisées?

- Oui
- Non

Nombre de JOURS de consommation au cours des 7 derniers jours?

Principale source de nourriture consommée

- dons sociaux (faits par la communauté / la famille ou des individus)
- propre production
- crédit / prêt
- parents / amis / voisins
- pêche / chasse / cueillette
- Dons humanitaires (faits par l'état et / ou des organisations)
- paiement du travail
- achat
- Autre

» Section 3.4 Moyens de l'exploitation

Pour chacun des actifs ci-dessous indiquez le nombre detenu par votre ménage

1.Charette
Mettez 0 si non disponible

2.Attelage
Mettez 0 si non disponible

3.Pulvérisateur
Mettez 0 si non disponible

4.Moulin à grain
Mettez 0 si non disponible

5.Pompe

Mettez 0 si non disponible

6. Appareil de tourney disque

Mettez 0 si non disponible

7. Cuisinière

Mettez 0 si non disponible

8. Bicyclette

Mettez 0 si non disponible

9. Mobylette

Mettez 0 si non disponible

10. Car

Mettez 0 si non disponible

11. Tracteur

Mettez 0 si non disponible

12. Maison au toit de chaume

Mettez 0 si non disponible

13. Maison en tuile

Mettez 0 si non disponible

14. Etang piscicole

Mettez 0 si non disponible

15. Autres actifs

- Oui
- Non

16. Autres actifs en nombre

» Section 3.5 Insecurite alimentaire

Aviez-vous assez de produits alimentaires pour couvrir les besoins du ménage durant les 12 derniers mois?

- Oui
- Non

Au cours du dernier mois, y a-t-il eu un moment où vous ou d'autres membres de votre ménage craigniez de ne pas avoir assez à manger à cause du manque d'argent ou d'autres ressources?

- Oui
- Non

Si oui, à quelle fréquence est-ce arrivé?

1 fois, 2 fois, etc. par mois

Au cours du dernier mois, y a-t-il eu un moment où vous ou d'autres membres de votre ménage avez été incapable de manger des aliments sains et nutritifs à cause du manque d'argent ou d'autres ressources?

- Oui
- Non

Si oui, à quelle fréquence est-ce arrivé?

1 fois, 2 fois, etc. par mois

Au cours du dernier mois, y a-t-il eu un moment où vous ou d'autres membres de votre ménage n'avez mangé que quelques types de nourriture en raison d'un manque d'argent ou d'autres ressources?

- Oui
- Non

Si oui, à quelle fréquence est-ce arrivé?
1 fois, 2 fois, etc. par mois

Au cours du dernier mois, y a-t-il eu un moment où vous ou d'autres membres de votre ménage avez dû sauter un repas parce qu'il n'y avait pas assez d'argent ou d'autres ressources pour acheter de la nourriture?

- Oui
- Non

Si oui, à quelle fréquence est-ce arrivé?
1 fois, 2 fois, etc. par mois

Au cours du dernier mois, y a-t-il eu un moment où vous ou d'autres membres de votre ménage avez mangé moins que vous ne le pensiez à cause d'un manque d'argent ou d'autres ressources?

- Oui
- Non

Si oui, à quelle fréquence est-ce arrivé?
1 fois, 2 fois, etc. par mois

Au cours du dernier mois, y a-t-il eu un moment où vous ou d'autres membres de votre ménage n'avez pas de nourriture en raison d'un manque d'argent ou d'autres ressources?

- Oui
- Non

Si oui, à quelle fréquence est-ce arrivé?
1 fois, 2 fois, etc. par mois

Au cours du dernier mois, y a-t-il eu un moment où vous ou d'autres membres de votre ménage avez eu faim mais n'avez pas mangé parce qu'il n'y avait pas assez d'argent ou d'autres ressources pour se nourrir?

- Oui
- Non

Si oui, à quelle fréquence est-ce arrivé?

1 fois, 2 fois, etc. par mois

Au cours du dernier mois, y a-t-il eu un moment où vous ou d'autres membres de votre ménage avez été sans manger pendant une journée entière à cause du manque d'argent ou d'autres ressources?

- Oui
- Non

Si oui, à quelle fréquence est-ce arrivé?

1 fois, 2 fois, etc. par mois

» Faire face aux pénuries alimentaires

Si vous avez fait face à une quelconque pénurie alimentaire ces 12 derniers mois, quelles sont les stratégies d'adaptation que vous avez utilisées? (pointer convenablement)

Selectionnez les mécanismes d'adaptation

- emprunt
- reduitrepas
- reduitcuis
- ventpatr
- empruntvoi
- Autre

Autre, à preciser.

Combien des mécanismes avez-vous séléctionnés?

» » Posez les questions suivant pour chacun des mécanismes d'adataption séléctionnés

Section 4. Progression des indicateurs de Pauvreté

1. Un membre du ménage a-t-il terminé au moins cinq années de scolarité?

- Oui
- Non

2. Est-ce que tous les membres du ménage âgés de 6 à 16 vont actuellement à une école ou d'un établissement d'enseignement reconnu officiellement?

- Oui
- Non

3. Y a-t-il un enfant d'âge scolaire qui ne fréquente pas l'école jusqu'à l'âge auquel il terminerait la classe 9?

- Oui
- Non

3. Nombre de chambres dans le ménage ?

______

4. Nombre d'hectares de terre cultivable dans le menage ?

- Moins d'un hectare
- 2 hectare à 5 hectares
- 7 hectares à 10 hectares
- 12 hectares à 15 hectares
- 17 hectares à 30 hectares
- 32 hectares à 60 hectares
- Plus de 60 hectares

5. Le chef / conjoint de sexe masculin peut-il lire et écrire une phrase simple en Français ou en Tshiluba?

- Non
- Anglais uniquement
- Français uniquement
- pas de chef / conjoint masculin
- Français et Tshiluba

6. La femme chef (la plus âgée) / conjointe peut-elle lire et écrire une phrase simple en Français ou en Tshiluba?

- Non
- Anglais uniquement
- Français uniquement
- pas de chef / conjoint masculin
- Français et Tshiluba

7. Quel est le matériau principal du sol de la résidence?

- En terre tassee
- Bois
- Ciment
- Briques
- Cailloux
- Carreaux
- Autre

8. Est-ce que votre ménage a hydro électricité ?

- Oui
- Non

8. Est-ce que votre ménage a l'énergie solaire ?

- Oui
- Non

9. Quel est le principal type de toilettes utilisé par le ménage?

- brousse
- latrine à fosse et non couverte
- latrine à fosse secrète
- Autre

9. Autre, à preciser.

____

10. Quelle est la principale source d'eau potable?

- ◯ Puits
- ◯ Ruisseau
- ◯ Forage
- ◯ Robinet
- ◯ Autre

10. Autre, à preciser.

____

11. Quel est le principal type de combustible utilisé pour cuisiner?

- ◯ Bois de chauffage collecté / offert, ou autre
- ◯ Bois de chauffage acheté, kérosène / paraffine / pétrole, charbon de bois, sciure / copeaux de bois, électricité ou ne cuit pas
- ◯ Autre

11. Autre, à preciser.

____

12. Votre ménage possède-t-il une radio ou des téléviseurs?

- ◯ Non
- ◯ Radio
- ◯ Télévision
- ◯ Radio et télévision

13. Votre ménage possède-t-il un fer à repasser? (électrique ou non électrique)

- ◯ Oui
- ◯ Non

14. Votre ménage possède-t-il des téléphones cellulaires?

- ◯ Oui
- ◯ Non

15. Le ménage possède-t-il une voiture ou un camion?

- ◯ Oui
- ◯ Non

Heure de clôture d'enquete

11:00
